# *Xanthoceras sorbifolium* Oil Attenuates Hyperlipidemia Through Dual Modulation of Gut Microbiota and Lipid Metabolites: Mechanistic Insights from Lipidomics and 16S rRNA Sequencing

**DOI:** 10.3390/metabo15050291

**Published:** 2025-04-25

**Authors:** Yameng Tao, Miaomiao Yao, Qi He, Xiaoyang Kang, Fangkai Shi, Xuan Hu, Zhiyun Meng, Hui Gan, Ruolan Gu, Yunbo Sun, Guifang Dou, Shuchen Liu

**Affiliations:** 1 School of Pharmaceutical Sciences, Anhui Medical University, Hefei 230032, China; 2245010880@stu.ahmu.edu.cn (Y.T.);; 2 Beijing Institute of Radiation Medicine, Beijing 100850, China; 3 College of Life Sciences, Hebei University, Baoding 071002, China; 4 School of Pharmacy, Henan University of Chinese Medicine, Zhengzhou 450046, China

**Keywords:** hyperlipidemia, *Xanthoceras sorbifolium* oil, gut microbiota, lipid metabolism, short-chain fatty acids

## Abstract

**Background/Objectives:** *Xanthoceras sorbifolium* oil (XSO), containing nervonic acid and unsaturated fatty acids (93%), exhibits lipid-lowering potential; yet, its mechanisms involving gut–liver crosstalk remain unclear. This study investigated XSO’s anti-hyperlipidemic effects and gut microbiota interactions. **Methods**: Forty-eight Sprague Dawley male rats were divided into: normal control (NC), high-fat diet (HFD), XSO prevention (XOP, 1.4 mL/kg pre-HFD), and XSO treatment (XOT, post-HFD). Serum lipids, fecal short-chain fatty acids (SCFAs), gut microbiota (16S rRNA), and lipidomics (UPLC-MS/MS) were analyzed after 12 weeks. **Results**: XOP significantly reduced serum total cholesterol (TC, 26.8%), triglycerides (TG, 35.9%), and low-density lipoprotein cholesterol (LDL-C, 45.9%) versus HFD (*p* < 0.05), while increasing high-density lipoprotein cholesterol (HDL-C, 7.98%). XOP showed enhanced hepatoprotection (AST↓ 32.6%, *p* < 0.01). Although XSO elevated fecal acetate (1.5-fold) and butyrate (1.3-fold), these changes lacked significance (*p* > 0.05). The analysis of gut microbiota showed that the pro-inflammatory *Coriobacteriaceae* and *Erysipelibacteriaceae* were reduced at the family level in the XOP group (*p* < 0.05). Lipidomics identified 69 differential metabolites: XSO downregulated atherogenic cholesteryl esters and triglycerides, upregulated six phosphatidylethanolamines, and modulated aberrant lysophosphatidylcholines. **Conclusions**: XSO alleviates hyperlipidemia through direct modulation of lipid metabolism pathways and suppression of pro-inflammatory gut microbiota. While its prebiotic potential warrants further validation, these findings highlight XSO as a functional dietary adjunct for improving lipid homeostasis and mitigating cardiovascular risks. XSO alleviates hyperlipidemia through direct modulation of lipid metabolism pathways and suppression of pro-inflammatory gut microbiota, while its prebiotic potential warrants further validation. These findings support XSO as a dietary adjunct for lipid homeostasis improvement, offering a nutritional strategy for early-stage cardiovascular risk management.

## 1. Introduction

Cardiovascular diseases (CVDs) constitute the foremost global health burden, responsible for 18.6 million deaths annually, with atherosclerotic cardiovascular disease (ASCVD) being the most common type [1,2]. Dyslipidemia, especially hyperlipidemia, is highly prevalent in adults worldwide and elevates ASCVD risk by 2-fold through dysregulated lipid metabolism [3]. However, CVDs mortality is expected to surge in the coming decades due to the aging of the global population [4,5]. Hyperlipidemia is characterized by elevated total cholesterol (TC), triglycerides (TG), and low-density lipoprotein cholesterol (LDL-C), accompanied by systemic imbalances including carbohydrate-lipid metabolism dysfunction, vascular wall thickening, and obesity-related comorbidities [6]. In addition, emerging lipidomic evidence identifies pathogenic lipid species, particularly lysophosphatidylcholine (LPC) and ceramides, as independent risk predictors of CVDs [7,8,9]. The escalating global prevalence of hyperlipidemia, driven by the widespread adoption of energy-dense diets and sedentary behaviors, underscores the compelling need for developing functional foods targeting lipidome remodeling through microbiota-metabolism crosstalk.

While statins remain first-line pharmacological interventions for hyperlipidemia, their long-term application is constrained by dose-dependent adverse effects such as hepatotoxicity and myopathy [10]. Notably, contemporary lipid metabolomics studies reveal that statins exhibit limited efficacy in modulating specific pathogenic lipid subclasses, including pro-inflammatory LPC and ceramide species associated with insulin resistance [11]. This therapeutic gap has spurred interest in nutrient-based interventions targeting systemic lipidomic remodeling. Functional foods enriched with bioactive components demonstrate cholesterol-lowering efficacy through dual regulation of glycerolipid metabolism and sphingolipid signaling pathways [12,13].

Emerging evidence implicates serum lysophospholipids (e.g., LPC and lysophosphatidylethanolamine (LPE)) as critical mediators in hyperlipidemia progression. These amphipathic molecules not only serve as biomarkers of lipid peroxidation but also directly impair endothelial function by activating G protein-coupled receptors (GPCRs) [14]. Concurrently, gut microbiota dysbiosis exacerbates metabolic syndromes through three lipid-centric mechanisms: (1) Bacterial β-glucuronidases deconjugate bile acids, altering hepatic glycerolipid synthesis; (2) Microbial-derived trimethylamine (TMA) promotes foam cell formation via LPC oxidation; (3) Pathobiont-enriched communities elevate ceramide production through sphingomyelinase activation [15,16,17,18]. Notably, dietary patterns have emerged as central modulators of the gut microbiota. Diets rich in plant-based foods, monounsaturated fats, and polyphenols—such as the Mediterranean diet—enhance microbial diversity and favor the growth of beneficial bacteria like Bifidobacterium and Faecalibacterium prausnitzii, which contribute to metabolic homeostasis via short-chain fatty acid (SCFA) production. In contrast, high-fat, Western-style diets disrupt microbial balance and promote inflammation [19]. Despite advances in understanding the lipid–microbiota axis, the interplay between dietary lipids (e.g., nervonic acid) and host–microbiota co-metabolism of glycerophospholipids remains unexplored, particularly in the context of serum lipidome specificity.

*Xanthoceras sorbifolium* Bunge, a drought-resistant plant endemic to northern China, has been traditionally utilized for cholesterol management and vascular protection [20,21]. *Xanthoceras sorbifolium* oil (XSO), extracted from its seeds, contains 93% unsaturated fatty acids—predominantly linoleic acid (C18:2, ~50%) and oleic acid (C18:1, ~30%)—and 2.95% nervonic acid, a rare component with neuroprotective properties [22,23,24,25,26]. Experimental studies confirm XSO’s hypolipidemic effects, potentially mediated by LDL-C reduction and antioxidant activity [27,28]. However, critical knowledge gaps persist regarding its influence on gut microbial ecology and systemic lipid metabolism, particularly the interplay between microbiota-derived metabolites and cholesterol regulation.

No prior studies have systematically investigated its impact on the gut microbiota-lipid metabolism axis. Here, we hypothesize that XSO supplementation ameliorates diet-induced hyperlipidemia by restoring microbial balance and modulating key lipid-regulating pathways. Utilizing a high-fat diet (HFD)-fed rat model combined with multi-omics approaches, this study aims to: Investigate the effects of various administration regimens of XSO on lipid profiles and hepatic steatosis in rats with hyperlipidemia induced by an HFD; Characterize gut microbiota remodeling and associated metabolite shifts via 16S rRNA sequencing and lipidomics. Elucidate mechanistic links between microbial changes (e.g., SCFAs) and host cholesterol regulation. At the same time, lipid metabolomics data can also inform future research efforts in establishing the causal relationships between specific lipid species and hyperlipemia. Our findings will provide translational insights into XSO as a functional food for metabolic syndrome management.

## 2. Materials and Methods

### 2.1. Materials

XSO was provided by Runsheng Agricultural Science and Technology Co., Ltd. (Hebei, China); high-fat feed was purchased from Ruidi Biotechnology (Shenzhen, China) Co., Ltd.; maintenance feed for rats and mice was purchased from Keao Xieli Feed Co., Ltd. (Beijing, China); physiological sodium chloride solution was purchased from Kelun Pharmaceutical Co., Ltd. (Sichuan, China); TG, TC, LDL-C, high-density lipoprotein cholesterol (HDL-C), homocysteine (HCY), alanine aminotransferase (ALT), and aspartate aminotransferase (AST) detection kits were purchased from Roche Diagnostics Products Co., Ltd. (Shanghai, China).

### 2.2. Animal Research

#### 2.2.1. Experimental Animals

A total of 48 male Sprague Dawley (SD) rats, obtained from Keyu Animal Science and Technology Co. Ltd., License No: SCXK (Beijing, China) 2022-0004, 8 weeks old and weighing about 180 g weight were used in this study. Prior to experimentation, all animals were acclimated under standard conditions (temperature: 25 °C ± 3 °C, humidity: 50% ± 5%, 12 h light/dark cycle) for a minimum of 7 days. Animals were monitored twice daily, and their health status was estimated by a general assessment of animal activity, food and water intake, external appearance, and absence of disease. Ethical approval for all experimental procedures was granted by the Ethics Committee of the Beijing Institute of Radiation Medicine (Approval No. IACUC-DWZX-2020-503), ensuring adherence to the principles of animal welfare.

#### 2.2.2. Experimental Design

**Figure 1:**
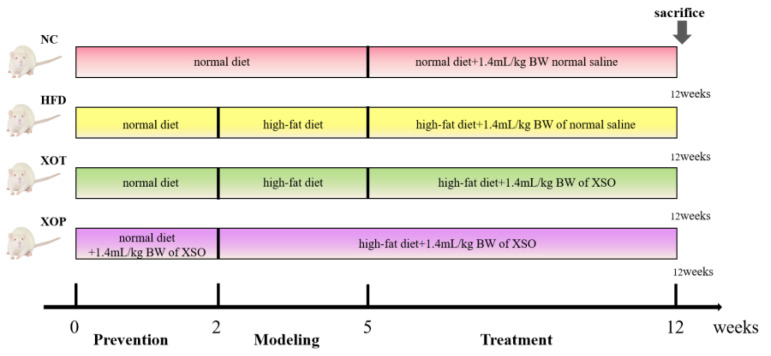
**Experimental design.** Abbreviations: NC: normal diet; HFD: high-fat diet; XOP: high-fat diet-induced model rats were orally gavaged with XSO for preventive purposes; XOT: high-fat diet-induced model rats were intragastrically administered with XSO for therapeutic intervention. (*n* = 12, per group).

Healthy adult male SD rats were acclimated to the laboratory environment, fed with standard maintenance feed, and provided with sterile water. After acclimation, the rats weighed 240 ± 10 g. At the end of the 7-day acclimation period, blood was collected from the orbital sinus of the rats, centrifuged at 3500 rpm for 15 min, and the supernatant was obtained. The contents of TC, TG, LDL-C, and HDL-C in the serum were detected using an automatic biochemical analyzer. The rats were randomly divided into 4 groups (12 rats in each group) based on the TC content, ensuring no significant difference in TC content among the groups. During the prevention period, each group was fed a normal diet. The prevention group began intragastric administration of 1.4 mL/kg of XSO. During the modeling period, the normal control group was fed a normal diet, while the other three groups were fed an HFD to establish a high-fat rat model. During the treatment period, the treatment group began intragastric administration of 1.4 mL/kg of XSO, and the model group was intragastrically administered the same dose of normal saline. The mode of administration for the four groups is presented in Figure 1. The experiment lasted for 12 weeks, and the body weight of the rats was measured weekly. To conduct 16S rRNA gene sequencing of the intestinal flora, fresh feces were collected in the 12th week. The rats were isolated in individual cages disinfected with 70% EtOH, and the excreted feces were immediately preserved in autoclaved Eppendorf tubes using sterile forceps and gloves [29]. Before sampling, the rats were fasted for 12 h without water deprivation. After anesthesia, blood was collected from the abdominal aorta, and each organ was dissected and weighed.

### 2.3. Body Weight Change and Organ Index in Rats

At the end of the seven-day adaptation period, the weight of the rats was weighed, and then the weight of the rats was recorded once a week. At the end of the 12-week experiment, the rats were anesthetized and killed after blood collection, dissected, and weighed for each organ, and the organ index was calculated according to the formula.

(1) Liver index = liver wet weight/body weight × 100

(2) Epididymal fat index = wet weight of epididymal fat/body weight × 100

(3) Perirenal fat index = perirenal fat wet weight/body weight × 100

### 2.4. Serum Biochemical Detection

Blood samples were collected weekly from the medial canthus of the rats under respiratory anesthesia. Serum was subsequently isolated by centrifugation at 3500 revolutions per minute for 15 min and was utilized for biochemical analysis. The activities of TC, TG, HDL-C, and LDL-C in the collected serum samples were analyzed using an automatic biochemical analyzer. At the conclusion of the twelfth week of the experiment, the rats were anesthetized, and blood was obtained from the abdominal aorta. The activities of TC, TG, HDL-C, LDL-C, HCY, ALT, and AST in the collected serum samples were analyzed using an automatic biochemical analyzer. Subsequently, the plasma atherosclerosis index and Castelli risk index were calculated in accordance with the formulas [30,31].

(4) $$\mathrm{Atherogenic}\;\mathrm{Index}\;(\mathrm{AI}):\;\mathrm{AI}=log\left(\frac{TG}{HDL}\right)$$

(5) $$\mathrm{Atherogenic}\;\mathrm{Coefficient}\;(\mathrm{AC}):\;\mathrm{AC}=\frac{TC-HDL}{HDL},$$

(6) $$\mathrm{Castelli}\;\mathrm{risk}\;\mathrm{index}\;(\mathrm{CRI}\text{-}I):\;\mathrm{CRI}\text{-}I=\frac{TC}{HDL},$$

(7) $$\mathrm{Castelli}\;\mathrm{risk}\;\mathrm{index}\;(\mathrm{CRI}\text{-}\mathrm{II}):\;\mathrm{CRI}\text{-}\mathrm{II}=\frac{LDL}{HDL}$$

### 2.5. Detection of SCFAs in Feces

The quantitative method for total SCFAs in feces was referred to in the aforementioned study [32,33]. Briefly, fecal samples of 30–60 mg were accurately weighed and then extracted with dichloromethane. After repeated extractions, derivatization was carried out using N,O-bis(trimethylsilyl) trifluoroacetamide (BSTFA). A 7890 gas chromatograph and 7000D mass spectrometer (Agilent Technologies, CA, USA) were employed, and chromatographic separation was performed using an HP-5ms capillary column (30 m × 0.25 mm × 0.25 μm) (Agilent Technologies) to analyze the organic supernatant. The fecal SCFA was calculated by the external standard method.

### 2.6. 16S rRNA Gene Sequencing Analysis

Two milliliters of rat feces were collected in 2 mL screw-cap microtubes and stored at −80 °C. The genomic DNA of the microbial community was extracted from the fecal samples using the CTAB method (Noble Bio, Zhejiang, China), following standardized procedures. The DNA concentration and purity were determined with a micro-volume spectrophotometer and verified by 1% agarose gel electrophoresis (Thermo Scientific, MA, USA). The hypervariable V3-V4 region of the bacterial 16S rRNA gene was amplified using the primer pair 341F (CCTAYGGGRBGCASCAG) and 806R (GGACTACNNGGGTATCTAAT). Equal amounts of PCR products were mixed based on their concentrations. After thorough mixing, the PCR products were purified using 2% agarose gel electrophoresis. The target bands were recovered using the gel recovery kit provided by QIAGEN (Hilden, Germany). The TruSeq^®^ DNA PCR-Free Sample Preparation Kit was employed for library construction. The constructed libraries were quantified using the Qubit/Agilent Bioanalyzer 2100 System/Q-PCR (CA, USA) and then sequenced on the NovaSeq 6000 (CA, USA). Library construction and sequencing were accomplished by Wuhan Maiwei Metabolic Biotechnology Co., Ltd. (Wuhan, China).

### 2.7. Serum Lipid Metabolomics Analysis

Blood samples were collected from the abdominal aorta and centrifuged at 3000 rpm for 15 min to isolate the serum. For lipid extraction, 1 mL of methyl tert-butyl ether:methanol (3:1, *v*/*v*) containing internal standards was added to 100 μL of serum and vortexed for 15 min. Then, 200 μL of water was added and vortexed for 1 min, followed by centrifugation at 12,000 rpm for 10 min at 4 °C. The supernatant was transferred, dried under vacuum, and reconstituted in 200 μL of acetonitrile:isopropanol (1:1, *v*/*v*). After vortexing and centrifugation (12,000 rpm, 3 min), the supernatant was subjected to UPLC-MS/MS analysis. Chromatographic separation was performed using an ExionLC™ AD UPLC system (SCIEX, MA, USA) equipped with a Thermo Accucore™ C30 column (2.6 μm, 2.1 mm × 100 mm, MA, USA). Mass spectrometric detection was carried out on a QTRAP^®^ 6500+ triple quadrupole mass spectrometer operating in Multiple Reaction Monitoring (MRM) mode.

Lipid species were identified and annotated based on retention time, parent–daughter ion pairs, and MS/MS fragmentation patterns using a self-built database (MWDB, MetWare Biotechnology, Wuhan, China). For quantification, class-specific internal standards were used, and lipid concentrations were calculated based on the ratio of analyte peak area to internal standard peak area, according to the following formula:

(8) X = 0.001 × R × c × F × V/m

Annotation: X is the concentration of the target lipid (nmol/mL), R is the area ratio of analyte to the internal standard, c is the internal standard concentration (μmol/L), F is the correction factor, V is the volume of extraction solvent (μL), and m is the sample volume (mL). This quantification approach is consistent with validated high-throughput lipidomics methods previously described [34].

### 2.8. Statistical Analysis

All results are expressed as mean ± standard deviation (SD). Statistical analyses were performed using GraphPad Prism 9 (GraphPad, USA). For the time-course assessment of serum lipid levels (1–11 weeks), a two-way analysis of variance (ANOVA) was conducted to evaluate the effects of treatment and time, followed by Dunnett’s multiple comparisons test, comparing each experimental group to the HFD group from week 6 onward. For endpoint measurements collected at the end of the experiment—including serum biochemical parameters, cardiovascular indices, body weight, organ weight, serum lipid metabolites, and fecal SCFAs—one-way ANOVA followed by Dunnett’s multiple comparisons test was applied, using the HFD group as the control. A *p*-value < 0.05 was considered statistically significant in all analyses. Regarding the data of the intestinal microbiota, the taxonomic composition at the phylum, family, and genus levels was depicted as bar charts. Diversity was evaluated through Chao1, observed_ASV, ACE, and Shannon indices. For lipidomics analysis, lipid identification was carried out by searching MS and MS/MS information in the Metware Metabolomics Database and HMDB. All the above data were processed in Metware Cloud (https://cloud.metware.cn) and GraphPad Prism 9 (GraphPad, CA, USA). The *p* value < 0.05 was regarded as statistically significant.

## 3. Results

### 3.1. Body Weight and Tissue Weight

**Figure 2:**
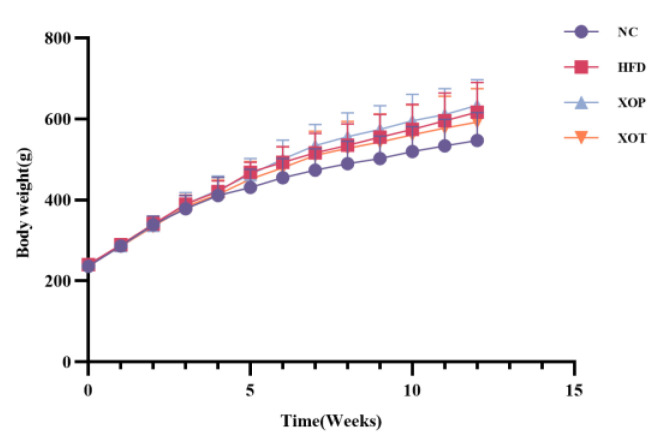
**The effect of different administration methods of*****Xanthoceras sorbifolium*****oil on the body weight of high-fat rats.** Abbreviations: NC: normal diet; HFD: high-fat diet; XOP: high-fat diet-induced model rats were orally gavaged with XSO for preventive purposes; XOT: high-fat diet-induced model rats were intragastrically administered with XSO for therapeutic intervention. Data are presented as mean ± standard deviation (SD), *n* = 12.

Upon the conclusion of the feeding research, the body weight, liver weight, perirenal fat weight, epididymal fat weight, spleen weight, and pancreas weight of the rats in the HFD group exhibited a significant increase when compared to those in the normal diet group. The spleen weight of the treatment group demonstrated a significant reduction. There was no significant variance in body weight between the XSO prevention group and the treatment group, in contrast to the rats in the HFD group, nor were there significant differences in liver weight, perirenal fat weight, epididymal fat weight, and pancreas weight. After 12 weeks of the feeding experiment, there was no significant difference in the weight of the kidney, testis, epididymis, and brain among the four groups. (Table 1 and Table 2, Figure 2).

### 3.2. Serum Biochemical Parameters and Cardiovascular Indices

**Figure 3:**
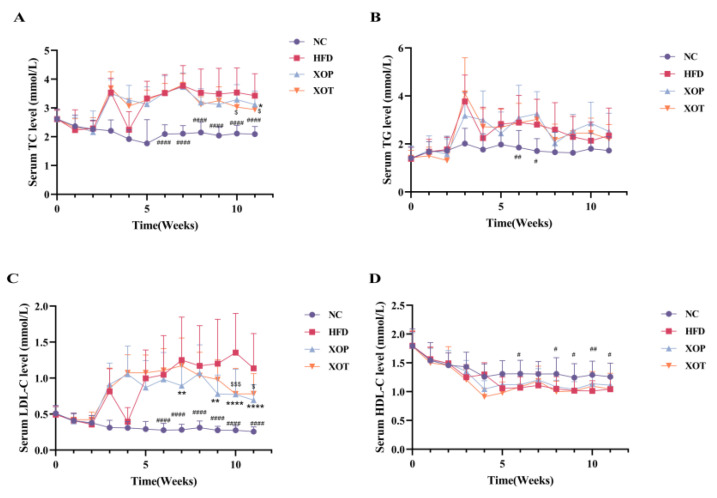
**Effects of XSO on the contents of (A) TC, (B) TG, (C) LDL-C, and (D) HDL-C in the serum of rats in each group per week (0–11 weeks).** Blood samples were collected from non-fasted rats to reflect postprandial lipid status. Data are presented as mean ± standard deviation (SD), *n* = 12. Statistical analysis was performed from week 6 onward using two-way ANOVA followed by Dunnett’s post hoc test, with the HFD group used as the reference. XOP group compared with the model group: **** *p* < 0.0001; ** *p* < 0.01; * *p* < 0.05; XOT group compared with the model group: ^$$$^ *p* < 0.001; ^$^ *p* < 0.05; NC group compared with the model group, ^####^ *p* < 0.0001; ^##^ *p* < 0.01; ^#^ *p* < 0.05.

**Figure 4:**
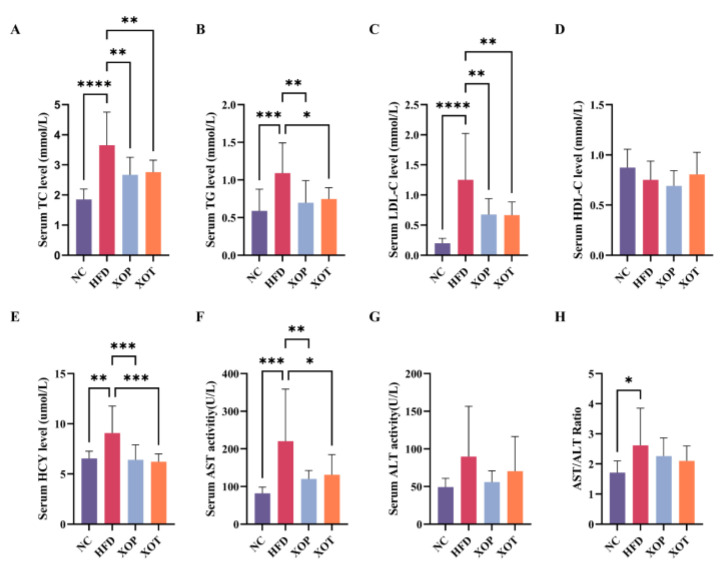
**Effects of XSO on serum biochemical indicators (at the end of the 12-week experiment).** Blood samples were collected after overnight fasting to assess fasting lipid metabolism. (**A**) Serum total cholesterol. (**B**) Serum triglyceride. (**C**) Serum low-density lipoprotein cholesterol. (**D**) Serum high-density lipoprotein cholesterol. (**E**) Serum homocysteine. (**F**) Serum aspartate aminotransferase. (**G**) Serum alanine aminotransferase. (**H**) Ratio of serum aspartate aminotransferase to alanine aminotransferase. Data are presented as mean ± standard deviation (SD), *n* = 12. Statistical differences were evaluated using one-way ANOVA followed by Dunnett’s multiple comparisons test. **** *p* < 0.0001; *** *p* < 0.001; ** *p* < 0.01; * *p* < 0.05.

At the end of the adaptation period, the serum lipid levels of rats in all groups were essentially at the same baseline. Compared with feeding a normal diet, feeding a high-fat diet for 5 weeks significantly increased plasma TC from 1.77 to 3.33 mmol/L, indicating the induction of a hypercholesterolemia model was successful, as 3.00 mmol/L was usually used as a cut-off value to classify hypercholesterolemia in rats. Over time, the levels of TC, TG, and LDL-C in both the prevention and treatment groups, which received intragastric administration of XSO, decreased to varying extents (Figure 3). After 12 weeks, compared with the normal control group (NC), the model group (HFD) showed significantly elevated levels of TC, TG, and LDL-C, while HDL-C decreased, albeit without statistical significance. Both the preventive and therapeutic groups treated with XSO significantly reduced TC, TG, and LDL-C levels in the model group and increased HDL-C content (Figure 4). The XOP group achieved reductions of 26.8%, 35.9%, and 45.9% in TC, TG, and LDL-C levels, respectively, within the HFD group (*p* < 0.01), while the XOT group demonstrated decreases of 24.5%, 31.6%, and 46.9%, respectively (*p* < 0.05). Furthermore, homocysteine levels were assessed across all groups at the conclusion of the 12th week. HCY levels were significantly elevated in the HFD group compared to the NC group. Concurrently, the hyperhomocysteinemia induced by the HFD was attenuated by 29.5% and 31.6% in the XOP and XOT groups, respectively. (*p* < 0.001) (Figure 4). Liver function index analysis revealed that, relative to the normal diet control group, serum ALT and AST levels increased in the HFD control group, indicative of potential liver injury or hepatotoxicity. Conversely, compared with the HFD group, serum AST levels decreased significantly in the XOP and XOT groups by 45.5% (*p* < 0.01) and 40.5% (*p* < 0.05), respectively, while ALT levels decreased by 37.7% and 21.6%, respectively, albeit without statistical significance (Figure 4). These findings suggest that XSO supplementation may protect against liver injury and enhance lipid metabolism in rats fed a high-fat diet.

The plasma atherosclerosis index (AI), atherogenic coefficient (AC), Castelli risk index I (CRI-I), and Castelli risk index II (CRI-II) were significantly associated with HFD exposure [35]. Significant alterations in cardiovascular indices were observed following prolonged consumption of XSO. Specifically, these indices were markedly elevated in the HFD group compared to the NC group (Table 3). Notably, both the plasma AI, AC, and CRI-II in the XOP and XOT groups treated with XSO decreased to levels comparable to those observed in the NC group (Table 3). These findings suggest that XSO may effectively mitigate the risk of arterial stiffness.

### 3.3. Fecal SCFAs

**Figure 5:**
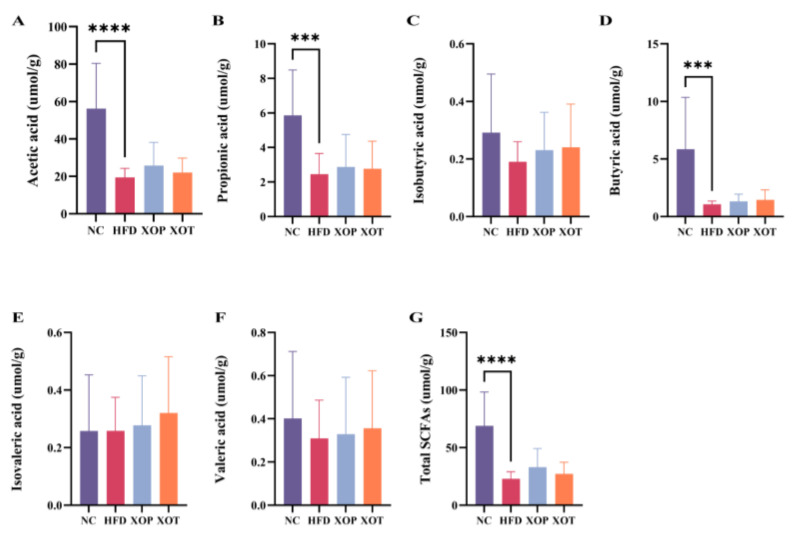
**Effects of XSO on the content of short-chain fatty acids in rat feces.** (**A**) Acetic acid. (**B**) Propionic acid. (**C**) Isobutyric acid. (**D**) Butyric acid. (**E**) Isovaleric acid. (**F**) Valeric acid. (**G**) Total SCFAs. Data are presented as mean ± standard deviation (SD), *n* = 10. Statistical differences were evaluated using one-way ANOVA followed by Dunnett’s multiple comparisons test. Compared with the control group, *** *p* < 0.001; **** *p* < 0.0001.

SCFAs are gut-derived metabolites produced in the large intestine, which participate in the modulation and regulation of cardiometabolic processes. GC-MS analysis demonstrated that an HFD markedly decreased the total SCFA content in the feces of normal control rats, as well as the concentrations of acetic acid, propionic acid, and butyric acid. In comparison to the HFD group, both the preventive and therapeutic groups supplemented with XSO exhibited increased levels of total SCFAs in feces, including acetic acid, propionic acid, butyric acid, isobutyric acid, valeric acid, and isovaleric acid. However, none of these differences reached statistical significance (*p* > 0.05), indicating that while XSO may exert a mild modulatory effect on SCFA production, the evidence remains insufficient to draw definitive conclusions (Figure 5).

### 3.4. Effects of XSO on Gut Microbial Diversity

**Figure 6:**
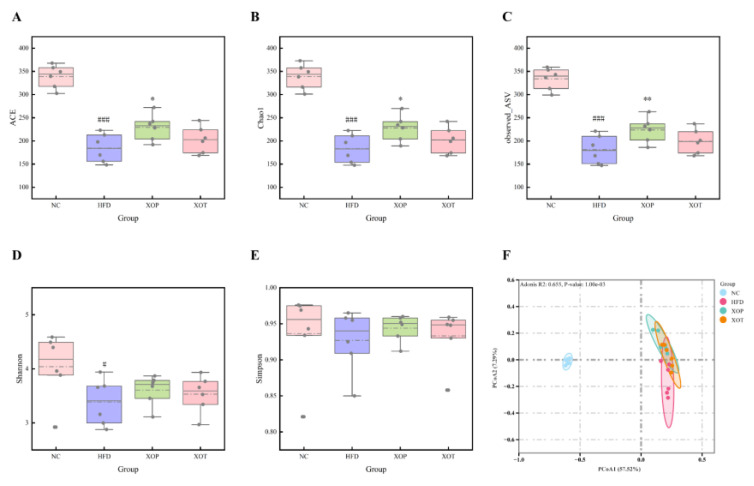
**Effects on intestinal flora diversity of rats in each group.** (**A**) ACE Species richness Index. (**B**) Chao1 species richness index. (**C**) observed_ASV index. (**D**) Shannon Diversity Index. (**E**) Simpson Diversity Index. (**F**) Beta-diversity by PCoA based on the Bray–Curtis index distance and permutational multivariate analysis of variance (PERMANOVA). Kruskal–Wallis test was used for alpha-diversity, *n* = 6. Compared with the blank control group, ^#^ *p* < 0.05; ^###^ *p* < 0.001; Compared with the high-fat model group, * *p* < 0.05; ** *p* < 0.01.

The development of hyperlipidemia is frequently associated with intestinal microbiota dysbiosis. Compared to the normal control diet, HFD feeding significantly reduced the alpha diversity of the gut microbiota, as evidenced by lower Shannon, observed ASV, Chao1, and ACE indices (Figure 6) (*p* < 0.05). Conversely, supplementation with XSO partially mitigated this decline; notably, the ACE index, Chao1 diversity index, and observed ASV in the prevention group were significantly higher than those in the model group. Principal coordinate analysis (PCoA) revealed that the model group and the normal group exhibited distinct clustering patterns, while the prevention and treatment groups showed significant shifts compared to the model group.

### 3.5. Effects of XSO on Intestinal Microbial Composition

**Figure 7:**
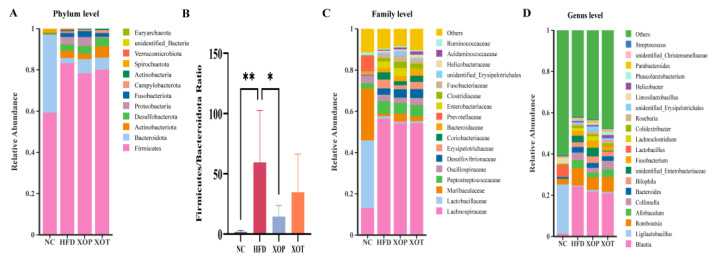
**(A–D) Analysis of gut microbiota composition.** (**A**) Bar plot analysis of the community at the phylum level. (**B**) Abundance of the *Firmicutes/Bacteroidetes* (F/B) ratio. Data are presented as mean ± standard deviation (SD), *n* = 6. Statistical analysis was performed using one-way ANOVA followed by Dunnett’s post hoc test. The difference in means was considered statistically significant at *p* < 0.05, ** *p* < 0.01; * *p* < 0.05. (**C**) Bar plot of community at the family level. (**D**) Bar plot of community at the genus level.

The gut microbiota was analyzed to assess its potential role in metabolic activation in SD rats following 12 weeks of intragastric administration of XSO, both prior to and after the establishment of an HFD model. The analysis of the gut microbiota composition revealed that *Firmicutes*, *Bacteroidetes*, *Actinobacteria*, *Desulfobacterota*, and *Proteobacteria* were the top 5 microbes at the phylum level (Figure 7A). Prophylactic administration of XSO significantly reduced the *Firmicutes* to *Bacteroidetes (*F/B*)* ratio, whereas administration of XS0 after modeling led to a decrease in F/B that did not reach statistical significance (Figure 7B). In the HFD group, the proportions of *Bacteroidetes* and *Firmicutes* were 3.79% and 82.16%, respectively, while in the XOP group, these values were 6.92% and 78.34%, respectively. The relative abundance of *Bacteroidetes* was also higher in the XOT group compared to the HFD group. The relative abundance at the family level revealed the bacterial composition changes. (Figure 7C). In detail, HFD increased *Coriobacteriaceae* from 0.01% in NC to 3.5% in HFD (*p* < 0.0001). However, the abundance of *Coriobacteriaceae* decreased to 1.9% in XOP (*p* < 0.05) and 3.0% in XOT compared to HFD. Similarly, the abundance of the *Erysipelotrichaceae* group increased by HFD from 1.0% in NC to 4.3% in HFD (*p* < 0.01) but decreased to 2.0% in XOP (*p* < 0.05) and 3.5% in XOT. To further investigate the differences in the intestinal microbiota among the HFD, XOP, and XOT groups, we analyzed the top 20 genera with significant changes. The identified genera included *Blautia*, *Romboutsia*, *Allobaculum*, and *Collinsella* (Figure 7D). The average relative abundance of *Blautia* was measured at 1.4%, 24.5%, 21.8%, and 20.7% in the NC, HFD, XOP, and XOT groups, respectively (n.s.). Equally, the *Romboutsia* genus showed relative abundances of 2.5%, 8.3%, 6.0%, and 7.2% across the groups (n.s.). *Allobaculum* showed lower relative abundances, with 0.2%, 3.9%, 2.2%, and 3.8% across the groups (n.s). Hyperlipidemia disrupted the balance of the intestinal microbiota by increasing the F/B ratio and reducing the abundance of beneficial bacteria. However, both the prevention and treatment groups restored this balance. Notably, the abundance of *Bacteroides* increased in both groups administered XSO via gavage, and this trend was negatively correlated with the development of host metabolic disorders [36].

### 3.6. Changes in Genus Microorganisms and Related Parameters

**Figure 8:**
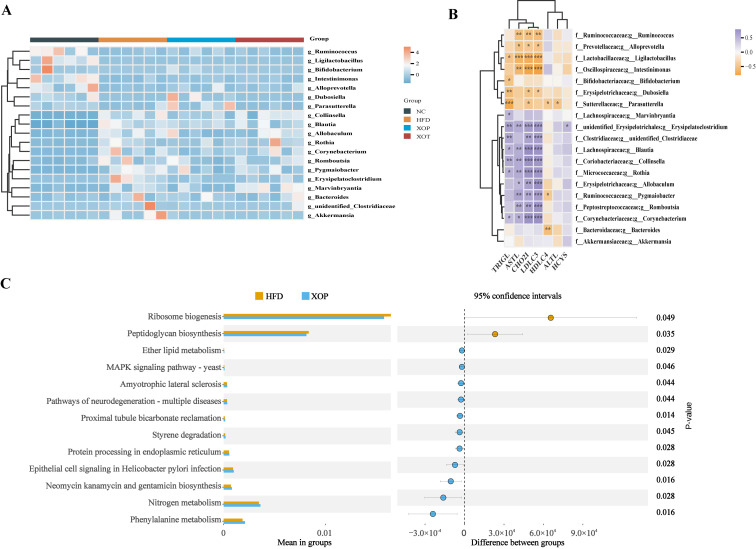
**(A–C) Analysis of gut microbiota composition.** (**A**) Heatmap of relative abundance at the genus level. (**B**) Spearman correlation heatmap of 19 specific genera and biochemical indicators. (**C**) Differential metabolic pathway enrichment in HFD and XOP (*t*-test, *p* < 0.05).

Spearman’s correlation analyses were conducted to correlate genus changes with parameters of cholesterol metabolism (Figure 8B). From the results shown in the figure, the genera *Erysipelatoclostridium*, *Rothia*, *Blautia*, *Romboutsia*, *Allobaculum*, and *Corynebacterium* exhibited positive correlations with TC, TG, LDL-C, and AST levels. These genera showed a decreasing trend in the preventive group administered with XSO (Figure 8A). Notably, *Parasutterella* and *Dubosiella*, their values in XOP show a slightly upward trend and show negative correlations with TC and TG levels. These findings suggest a bidirectional interaction between blood lipid profiles and gut microbiota, highlighting the beneficial role of XSO in modulating gut microbial composition in individuals with hyperlipidemia.

### 3.7. Enrichment Analysis of Microbial Metabolic Pathways

The metagenomic function prediction analysis conducted using PICRUSt2 for the HFD group and the XOP group revealed significant differences in the enrichment levels of metabolic pathways between the two groups (*t*-test, *p* < 0.05) (Figure 8C). Specifically, the HFD group exhibited enriched metabolic pathways associated with ribosome biosynthesis and peptidoglycan biosynthesis (95% confidence interval did not include zero, *p* < 0.05), which may indicate exacerbated bacterial proliferation and inflammatory responses. In contrast, preventive interventions appeared to restore metabolic equilibrium and mitigate inflammation risk by upregulating host ether lipid metabolism and phenylalanine metabolism, as well as inhibiting Helicobacter pylori-related signaling pathways (all 95% confidence intervals did not include zero, *p* < 0.05).

### 3.8. Serum Lipid Metabolism

**Figure 9:**
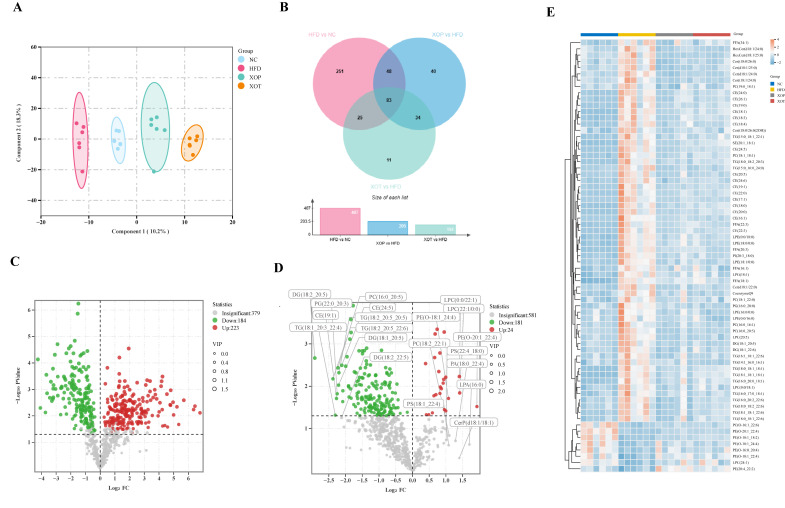
**(A–E) Lipidomics analysis.** (**A**) OPLS-DA score plots of the four groups. (**B**) Venn diagram and bar chart for evaluating the therapeutic effects of XSO on hyperlipidemic rats in the prevention and treatment groups. (**C**) Volcano plot of differentially expressed lipid metabolites between the HFD group and the NC group. (**D**) Volcano plot of differentially expressed lipid metabolites between the XOP group and the HFD group. (**E**) 69 serum lipid metabolites improved in the prevention and treatment groups. *n* = 6.

A quantitative lipidomics approach was employed to investigate serum lipid metabolites in rats under normal diet, HFD, and those receiving preventive or therapeutic administration of XSO. UPLC-MS/MS analysis of the four groups revealed distinct metabolic profiles through OPLS-DA score plots, indicating that both preventive and therapeutic administration of XSO could modulate HFD-induced alterations in serum lipid metabolites (Figure 9A). Venn diagram analysis demonstrated stronger regulatory effects of preventive administration, involving 205 differential metabolites (including 131 potential biomarkers), compared to 153 altered metabolites in the therapeutic group, over half of which were associated with hyperlipidemia risk (Figure 9B). Using screening criteria of VIP > 1 and *p* < 0.05, 181 upregulated and 24 downregulated metabolites were identified in the XOP group versus the HFD group (Figure 9D). Notably, 103 differential metabolites in the XOP group significantly ameliorated HFD-induced metabolic changes (Figure S1), with diglycerides (DG), cholesteryl esters (CE), triglycerides (TG), and phosphatidylcholines (PC) being predominantly downregulated in the preventive group, while LPC and phosphatidylethanolamines (PE) were upregulated.

### 3.9. Enrichment Pathway Analysis of Serum Lipid Metabolites

**Figure 10:**
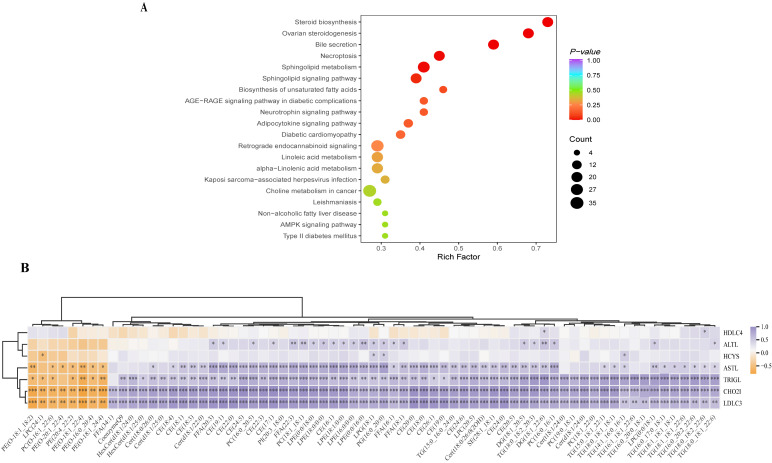
**(A,B) Lipidomics analysis.** (**A**) Metabolic pathway analysis of potential biomarkers. (**B**) Correlation analysis of 69 differential metabolites and biochemical indicators, with differences in means being statistically significant at *p* < 0.05,*** *p* < 0.001; ** *p* < 0.01; * *p* < 0.05; *n* = 6.

Next, KEGG analysis was performed around the lipid metabolites that were significantly changed after prophylactic gavage of XSO compared with the high-fat model group. KEGG pathway enrichment analysis of lipid metabolites revealed that preventive supplementation with XSO exerts regulatory effects on multiple metabolic pathways in HFD-induced hyperlipidemic rats, including steroid biosynthesis, bile secretion, necroptosis, sphingolipid metabolism, sphingolipid signaling pathway, biosynthesis of unsaturated fatty acids, and the AGE-RAGE signaling pathway in diabetic complications (Figure 10A).

### 3.10. Changes in Serum Lipid Metabolites and Related Parameters

The study on hyperlipidemia-associated metabolites was conducted using Pearson correlation analysis, which revealed significant correlations between serum lipid metabolism and hyperlipidemia-related physiological parameters. Specifically, glycerolipids (such as triglycerides and diglycerides) and sterol esters (CE) showed positive correlations with serum concentrations of TC, TG, LDL-C, and AST. In contrast, six PEs, LPC (24:1), and PC (O-16:1_22:6) exhibited negative correlations with these parameters. After treatment with XSO, the levels of these glycerophospholipids (GP) increased, a trend opposite to the changes observed in serum lipid profiles and ALT/AST levels. Although distinct lipid classes displayed divergent trends, the correlation analysis confirmed that glycerolipids (including TG and DG) and CEs were positively associated with TG, TC, and LDL-C (Figure 10B).

## 4. Discussion

Hyperlipidemia, as a disease closely associated with metabolic disorders, is influenced by multiple factors in its development. At the etiological level, exogenous factors primarily include unhealthy lifestyles such as an HFD and physical inactivity, while endogenous factors involve genetic susceptibility, particularly in individuals with a family history of lipid metabolism-related gene mutations [37]. Notably, overweight/obesity is not only an independent risk factor but also a critical pathological link that exacerbates lipid metabolism disorders by inducing insulin resistance [38,39]. Pathophysiological studies reveal that the core mechanism lies in the disruption of lipid metabolism homeostasis [40]. Recent research further demonstrates that gut microbiota dysbiosis exacerbates dyslipidemia through the “gut–liver axis” regulatory mechanism, which affects bile acid metabolism and short-chain fatty acid synthesis [41,42]. This discovery provides new therapeutic targets for disease intervention.

Polyunsaturated fatty acids (PUFAs), particularly ω-3 fatty acids derived from plant seed oils and fish oil, have been found to be beneficial for cardiovascular disease-related morbidity [43]. Micronutrients such as phytosterols and polyphenols are also natural lipid-lowering components. XSO is rich in PUFAs, phytosterols, and polyphenols, but its potential to regulate blood lipids remained unexplored. In this study, the mechanisms underlying XSO’s modulation of gut microbiota and lipid metabolism in hyperlipidemic rats were investigated using omics analyses. The results demonstrated that XSO effectively reduced serum levels of TC, TG, LDL-C, HCY, and AST (Figure 4). Similarly, different administration modes of XSO in hyperlipidemic model rats led to varying degrees of reversal in serum lipid profiles (Figure 9 and Figure 10). Dietary supplementation with XSO reduced fecal SCFA content (Figure 5) and modulated the composition of gut microbiota (Figure 7 and Figure 8). These findings indicate that XSO protects cardiovascular health by lowering plasma TC, TG, LDL-C, HCY, and AST levels while regulating gut microbiota and improving lipid metabolism. This study contributes to the evaluation of diverse plant seed oils in alleviating hyperlipidemia.

Long-term high-fat diets typically induce host obesity and hyperlipidemia by transferring TG from peripheral fat and promoting fat accumulation. The results of this study showed that the average body weight of hyperlipidemic rats increased by 15.53% compared to the NC group. After feeding hyperlipidemic rats with XSO via different administration methods, no significant effect on body weight was observed. This finding is consistent with previous reports that additional supplementation of peony seed oil on an HFD did not significantly alter body weight or fat content in golden Syrian hamsters [29].

Dyslipidemia is intricately linked with metabolic diseases, characterized by elevated levels of TC, LDL-C, and reduced levels of HDL-C [44]. Our study demonstrated that supplementation with XSO significantly decreased serum levels of TC, TG, and LDL-C in rats, while also improving liver function parameters. Notably, the preventive group exhibited more pronounced effects. When liver cells are damaged, serum levels of AST and ALT, markers of hepatic injury, increase [45]. XSO intervention markedly reduced AST levels, although its effect on ALT was less significant. HCY serves as a critical biomarker with multiple pathological implications in clinical evaluation [46]. Research has established a significant positive correlation between serum HCY concentration and coronary atherosclerotic plaque burden. Its dynamic changes not only effectively reflect the severity of coronary artery lesions but have also been confirmed as a sensitive early warning indicator of vascular endothelial injury [47]. Intervention with XSO significantly lowered serum HCY levels.

This study elucidated the anti-hyperlipidemic and anti-HCY properties of XSO, highlighting its significant potential in cardiovascular health protection and contributing to the development of XSO as a functional lipid-regulating agent. In this investigation, we observed marked alterations in serum lipid metabolism following XSO administration in rats fed HFD. A total of 69 lipid species were identified as potential biomarkers, primarily encompassing sterol esters, glycerides, glycerophospholipids, fatty acyls, sphingolipids, and isoprenoid esters. Lipid metabolism disorders are pathologically linked to hyperlipidemia, with lipid accumulation in metabolic organs—characterized by elevated levels of TG, DG, and CE—disrupting metabolic balance and contributing to insulin resistance, diabetes, and cardiovascular risk [48,49]. Notably, XSO exerted a regulatory effect on several lipid classes. In both the XOP and XOT groups, 13 TGs and 16 CEs were significantly downregulated, while 6 PEs were upregulated. Additionally, XSO modulated 34 other lipid species, including ceramides, DGs, and PCs, which are known to be involved in lipid signaling, inflammation, and membrane dynamics (Table S1). Beyond quantitative changes, the biological implications of these lipid alterations are noteworthy. Elevated PEs, particularly plasmalogen-type PE(P), may reflect adaptive membrane remodeling or increased autophagic flux; however, excessive accumulation could aggravate organelle stress and inflammation. Reduced CE levels may indicate enhanced cholesterol clearance or altered esterification, potentially alleviating lipid toxicity. Downregulation of TGs, especially medium-chain species, is suggestive of improved hepatic lipid handling and insulin sensitivity. Moreover, shifts in PCs and LPEs point to modulation of lipid transport and inflammatory signaling pathways [34,50,51]. Together, these lipidomic responses imply that XSO not only mitigates lipid accumulation but also reprograms key lipid regulatory networks involved in metabolic homeostasis. The XOP group exhibited broader improvements in lipid profiles than the XOT group, suggesting that early intervention with XSO may confer superior benefits in regulating lipid metabolism and preventing lipid-induced metabolic disturbances.

Based on our research findings, we have demonstrated that prophylactic supplementation with XSO can modulate multiple metabolic pathways in high-fat-diet-induced hyperlipidemic rats. These pathways include steroid biosynthesis, bile acid secretion, necroptosis, sphingolipid metabolism, sphingolipid signaling, and unsaturated fatty acid biosynthesis. This evidence suggests that XSO may represent a promising therapeutic option for lipid metabolism disorders associated with hyperlipidemia.

Based on the improvement of metabolites related to hyperlipidemia, Spearman correlation analysis confirmed that there is a significant correlation between serum lipid metabolism and physiological parameters of hyperlipidemia. For example, triglycerides and cholesterol esters are positively correlated with TC, TG, LDL-C, ALT, and AST concentrations (*p* < 0.05). After intervention with XSO, the content of LPC (including LPC (0:0/18:1) and LPC (20:5)) decreased, which is consistent with the trend of changes in physiological indicators (blood lipids, alanine transaminase, and aspartate transaminase levels) (Figure 4, Table S1). Meanwhile, fruit oil intervention significantly reversed the interference of HFD on GP metabolism. Although the change trends of different categories of GP vary, correlation analysis indicates that GPs (including LPC (20:5), LPC (0:0/18:1), LPE (18:1/0:0), LPE (0:0/18:0), etc.) and fatty acyls (FFA (16:1), FFA (18:1), FFA (20:3), FFA (34:1), and FFA (22:3)) are positively correlated with TC, TG, and LDL-C. Further research on differential lipid metabolites can be conducted to determine whether they can be used as therapeutic targets to prevent the development of hyperlipidemia or obesity.

The gut microbiota and its metabolites exert direct and indirect influences on hyperlipidemia through multiple mechanisms. Alterations in the composition of the gut microbiota have been noted in individuals with hyperlipidemia [39]. Our study demonstrated that the administration of XSO promoted intestinal health in hyperlipidemic rats. At the phylum level, the key bacterial groups involved were primarily *Bacteroidetes* and *Firmicutes*. The ratio of F/B is considered an indicator of intestinal microbial balance in metabolic diseases [49]. The intestinal flora disorder in the XOP group and the XOT group was improved to varying degrees, and the difference between the XOP group and the HFD group was statistically significant (*p* < 0.05).

The intestinal microbiome may influence the pathogenesis of cardiovascular diseases in hosts by producing microbial metabolites such as SCFAs, secondary bile acids, and lipopolysaccharides (LPS) [52,53,54,55,56]. In various species models, the *Coriobacteriaceae* and *Erysipelotrichaceae* families have been repeatedly reported to be closely associated with dyslipidemia [57]. Specifically, members of the *Coriobacteriaceae* family are closely related to plasma levels of Non-HDL-C and TG in hamsters [58]. The *Erysipelotrichaceae* family is linked to fat accumulation in human livers [59], and studies have shown that an *unclassified_f_Erysipelotrichaceae* is directly related to the cholesterol synthesis regulator SREBP1 [29]. Spearman correlation analysis indicates that genus microorganisms such as *g_Erysipelatoclostridium* and *g_Corynebacterium* are positively correlated with TC, TG, AST, and LDL-C. These genera-level *bacteria* are more abundant in the model group, and their levels are reduced by prophylactic administration. In this regard, a decrease in the abundance of *f_Coriobacteriaceae* and *f_Erysipelotrichaceae* associated with feeding XSO may contribute to the lipid and cholesterol-lowering activity of XSO observed in the serum.

This study employed PICRUSt2 functional prediction analysis to explore the differential effects of HFD and preventive interventions on the metabolic pathways of the gut microbiota. The HFD group showed significant enrichment in ribosome biosynthesis and peptidoglycan biosynthesis pathways. Ribosomes, essential for microbial protein synthesis, were upregulated, potentially promoting rapid gut microbiota proliferation, particularly the expansion of pathogenic bacteria such as Firmicutes. Increased peptidoglycan biosynthesis may exacerbate host inflammation through direct pro-inflammatory effects and disruption of the intestinal barrier [60]. In contrast, the prevention group exhibited activation of ether lipid metabolism and phenylalanine metabolism pathways. Ether lipids, key components of cell membrane phospholipids, enhance membrane stability and antioxidant functions, which may help restore metabolic balance and counteract the negative effects of HFD [61].

Although SCFAs are widely recognized for their beneficial roles in regulating lipid metabolism, inflammation, and vascular function [62,63], the increases observed in SCFA levels following XSO administration in this study did not reach statistical significance (Figure 5). While both preventive and therapeutic groups showed upward trends in fecal SCFA concentrations relative to the HFD group, these differences (*p* > 0.05) limit the strength of interpretation. Therefore, it is not possible to conclude that XSO exerts a definitive effect on SCFA production under the current experimental conditions. These findings suggest only a potential modulatory role of XSO on gut microbial metabolism, which warrants further investigation using larger cohorts or complementary microbiome analyses. In light of this, any SCFA-related benefits should be interpreted cautiously, and have been accordingly downplayed in the present discussion.

This study elucidates that the XSO diet not only regulates serum lipid metabolites but also modulates the composition and metabolic activities of the intestinal microbiota. Observations indicate that dietary fats with varying fatty acid compositions can influence plasma lipid profiles [64]. Recent studies have demonstrated that different types of dietary fats can alter serum lipid content and the composition of the intestinal microbiota to differing extents [65,66]. Further investigation into the effects of individual saturated, monounsaturated, polyunsaturated ω-6 and ω-3 fatty acids, as well as trace nutrients in vegetable oils, on lipid metabolism will hold significant importance.

While this study provides valuable insights into the lipid-lowering effects of XSO and its potential interaction with gut microbiota, several limitations should be acknowledged. First, although 16S rRNA sequencing and functional prediction analyses suggest that XSO modulates gut microbial composition and associated metabolic pathways, causality has not been established. Validation using germ-free animals or fecal microbiota transplantation (FMT) is necessary to confirm whether the observed lipid-modulating effects are truly microbiota-dependent. Second, no antibiotic-treated control group was included to assess XSO’s effects in the absence of gut microbiota, which limits the ability to fully separate host-directed effects from microbiota-mediated ones. Third, while XSO may influence SCFA production, the study lacked a positive dietary control known to enhance SCFAs (e.g., inulin), which would have helped benchmark and contextualize the microbiota-mediated effects of XSO. Future studies incorporating these models and controls are warranted to further dissect the microbiota-dependent mechanisms and compare the efficacy of XSO with known SCFA-enhancing dietary interventions.

## 5. Conclusions

Both preventive and therapeutic administration of XSO alleviated hyperlipidemia in rats, as evidenced by significantly reduced levels of TC, TG, and LDL-C in hyperlipidemic rats compared with the model group. Meanwhile, XSO decreased cardiovascular indices such as the atherogenic coefficient, cardiac risk ratio, and cardioprotective index, suggesting its potential to mitigate cardiovascular risk. Additionally, XSO significantly decreased serum HCY levels, suggesting its potential as an effective dietary regulator for managing high homocysteine levels. Furthermore, preventive administration of XSO markedly improved liver function, as indicated by a 32.6% reduction in AST levels (*p* < 0.01). Moreover, preventive administration of XSO significantly suppressed the abundance of pro-inflammatory intestinal flora families *Coriobacteriaceae* and *Erysipelotrichaceae* (*p* < 0.05), highlighting its potential role in modulating gut–liver interactions. Mechanistic studies revealed that lipidomics analysis demonstrated XSO’s ability to directly inhibit atherosclerotic metabolic products, such as CE 20:5 (*p* < 0.05), and regulate the steroid biosynthesis pathway, thereby confirming that its lipid-lowering effects are primarily mediated through metabolic pathway regulation. Although fecal SCFAs, including acetate, exhibited an upward trend (increased by 1.5-fold), these changes did not reach statistical significance (*p* > 0.05), indicating that the therapeutic efficacy of XSO is predominantly driven by lipid homeostasis regulation rather than relying on alterations in the intestinal microbiota. In conclusion, XSO, as a dietary supplement, exhibits promising application prospects for the early management of hyperlipidemia and holds research potential for hyperhomocysteinemia. However, further research is required to test optimal dosages and preparation forms of XSO.

## Figures and Tables

**Table 1:** **Effects of*****Xanthoceras sorbifolium*****oil on Body Weight and Hepatic and Adipose Tissue Parameters in SD Rats** (Data are presented as mean ± standard deviation (SD), *n* = 12.).

Group	NC	HFD	XOP	XOT
weight/g	505.42 ± 63.66	583.92 ± 74.2 *	597.83 ± 63.49	566.58 ± 78.06
Liver Weight (g)	12.15 ± 2.17	20.1 ± 3.93 ****	20.3 ± 3.71	19.21 ± 3.26
Perirenal Adipose (g)	8.4 ± 4.07	21.99 ± 9.22 ***	20.5 ± 5.37	17.46 ± 9.06
Epididymal Adipose (g)	9.08 ± 3.22	15.74 ± 6.36 *	18.01 ± 5.46	16.55 ± 8.14
Liver Index (%)	2.4 ± 0.43	3.44 ± 0.67 ***	3.4 ± 0.62	3.39 ± 0.57
Perirenal Adipose Index (%)	1.66 ± 0.8	3.77 ± 1.58 ***	3.43 ± 0.9	3.08 ± 1.6
Epididymal Adipose Index (%)	1.76 ± 0.44	2.62 ± 0.74 *	2.96 ± 0.66	2.81 ± 1.02

Data are presented as mean ± standard deviation (SD), n = 12. Statistical differences were evaluated using one-way ANOVA followed by Dunnett’s multiple comparisons test. Compared with the blank group, ****: *p* < 0.0001; ***: *p* < 0.001; *: *p* < 0.05. Abbreviations: NC: normal diet; HFD: high-fat diet; XOP: high-fat diet-induced model rats were orally gavaged with XSO for preventive purposes; XOT: high-fat diet-induced model rats were intragastrically administered with XSO for therapeutic intervention.

**Table 2:** **Effects of XSO on organ parameters in SD rats**.

Group	NC	HFD	XOP	XOT
Kidney (g)	3.21 ± 0.44	3.1 ± 0.49	3.21 ± 0.44	3.1 ± 0.49
Epididymis (g)	0.79 ± 0.06	0.84 ± 0.18	0.76 ± 0.11	0.83 ± 0.05
Testis (g)	3.85 ± 0.51	3.85 ± 0.28	3.63 ± 0.47	3.43 ± 0.3 ^#^
Brain (g)	1.92 ± 0.09	1.81 ± 0.18	1.78 ± 0.3	1.9 ± 0.2
Spleen (g)	0.95 ± 0.16	1.25 ± 0.41 *	1 ± 0.18	0.98 ± 0.24 ^#^
Pancreas (g)	1.06 ± 0.58	2.12 ± 0.63 ***	2.02 ± 0.54	1.89 ± 0.85
Aorta(heart) (g)	1.91 ± 0.26	1.64 ± 0.34 *	1.67 ± 0.22	1.55 ± 0.18
Thymus (g)	0.42 ± 0.12	0.55 ± 0.44	0.35 ± 0.11	0.32 ± 0.07

Data are presented as mean ± standard deviation (SD), *n* = 12. Statistical differences were evaluated using one-way ANOVA followed by Dunnett’s multiple comparisons test. Compared with the model group, ^#^: *p* < 0.05; compared with the blank group, ***: *p* < 0.001; *: *p* < 0.05.

**Table 3:** **Effects of XSO on the risk index**.

Group	AI	AC	CRI-I	CRI-II
NC	−0.20 ± 0.15	1.14 ± 0.14	2.14 ± 0.14	0.23 ± 0.08
HFD	0.15 ± 0.15 ^####^	4.12 ± 1.9 ^####^	5.12 ± 1.90 ^####^	1.83 ± 1.26 ^####^
XOP	−0.02 ± 0.09 *	2.91 ± 0.78 *	3.91 ± 0.78 *	1.03 ± 0.43 *
XOT	−0.03 ± 0.15 **	2.59 ± 1.01 **	3.59 ± 1.01 **	0.90 ± 0.50 **

Data are presented as mean ± standard deviation (SD), *n* = 12. Statistical differences were evaluated using one-way ANOVA followed by Dunnett’s multiple comparisons test. Compared with the model group: ** *p* < 0.01; * *p* < 0.05; compared with the blank group, ^####^ *p* < 0.0001.

## Data Availability

The original contributions presented in the study are included in the article/Supplementary Materials; further inquiries can be directed to the corresponding authors.

## Supplementary Materials

The following supporting information can be downloaded at https://www.mdpi.com/article/10.3390/metabo15050291/s1, Figure S1: Heatmap of 103 differential serum lipid metabolites in four groups; Figure S2: Violin plots of 69 differential serum lipid metabolites in four groups; Table S1: Changes in differential lipid metabolites in the sera of the four groups.

## Abbreviations

The following abbreviations are used in this manuscript:

XSO	*Xanthoceras sorbifolium* Oil
CVDs	Cardiovascular diseases
ASCVD	Atherosclerotic cardiovascular disease
TMA	Trimethylamine
SD	Sprague Dawley
HFD	High-fat diet
TC	Total Cholesterol
TG	Triglyceride
LDL-C	Low-density lipoprotein cholesterol
HDL-C	High-density lipoprotein cholesterol
AI	Atherogenic index
AC	Atherogenic coefficient
HCY	Homocysteine
AST	Aspartate amino transferase
ALT	Alanine aminotransferase
BSTFA	N,O-Bis(trimethylsilyl) trifluoroacetamide
LPS	Lipopolysaccharides
LPC	Lysophosphatidylcholine
LPE	Lysophosphatidylethanolamine
CE	Cholesterol ester
PC	Phosphatidylcholine
DG	Diglyceride
FFA	Fatty acid
PUFAs	Polyunsaturated fatty acids
SCFAs	Short-chain fatty acids
