# Supplementary material for: Xanthoceras sorbifolium Oil Attenuates Hyperlipidemia Through Dual Modulation of Gut Microbiota and Lipid Metabolites: Mechanistic Insights from Lipidomics and 16S rRNA Sequencing
Source: Metabolites. 2025 Apr 25;15(5):291. doi: 10.3390/metabo15050291 (PMC12113417; doi:10.3390/metabo15050291)
Supplement: Supplementary file 1 [file metabolites-15-00291-s001.zip › metabolites-3601362-supplementary.pdf]

Supplementary Material

***Xanthoceras sorbifolium* Oil Attenuates Hyperlipidemia through Dual Modulation of Gut Microbiota and Lipid Metabolites: Mechanistic Insights from Lipidomics and 16S rRNA Sequencing**

Table of Contents

Supplementary Tables S1

- Changes in differential lipid metabolites in the sera of the four groups

Supplementary Figures S1-2

- Heatmap of 103 differential serum lipid metabolites in four groups
- Violin plots of 69 differential serum lipid metabolites in four groups

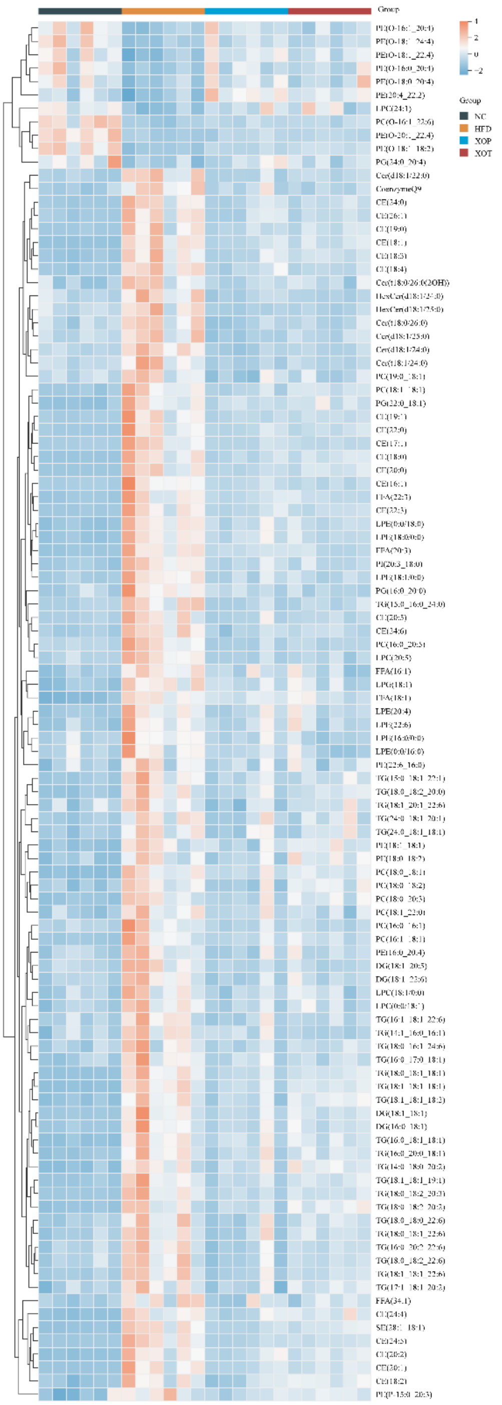

Figure S1. Heatmap of 103 differential serum lipid metabolites in four groups

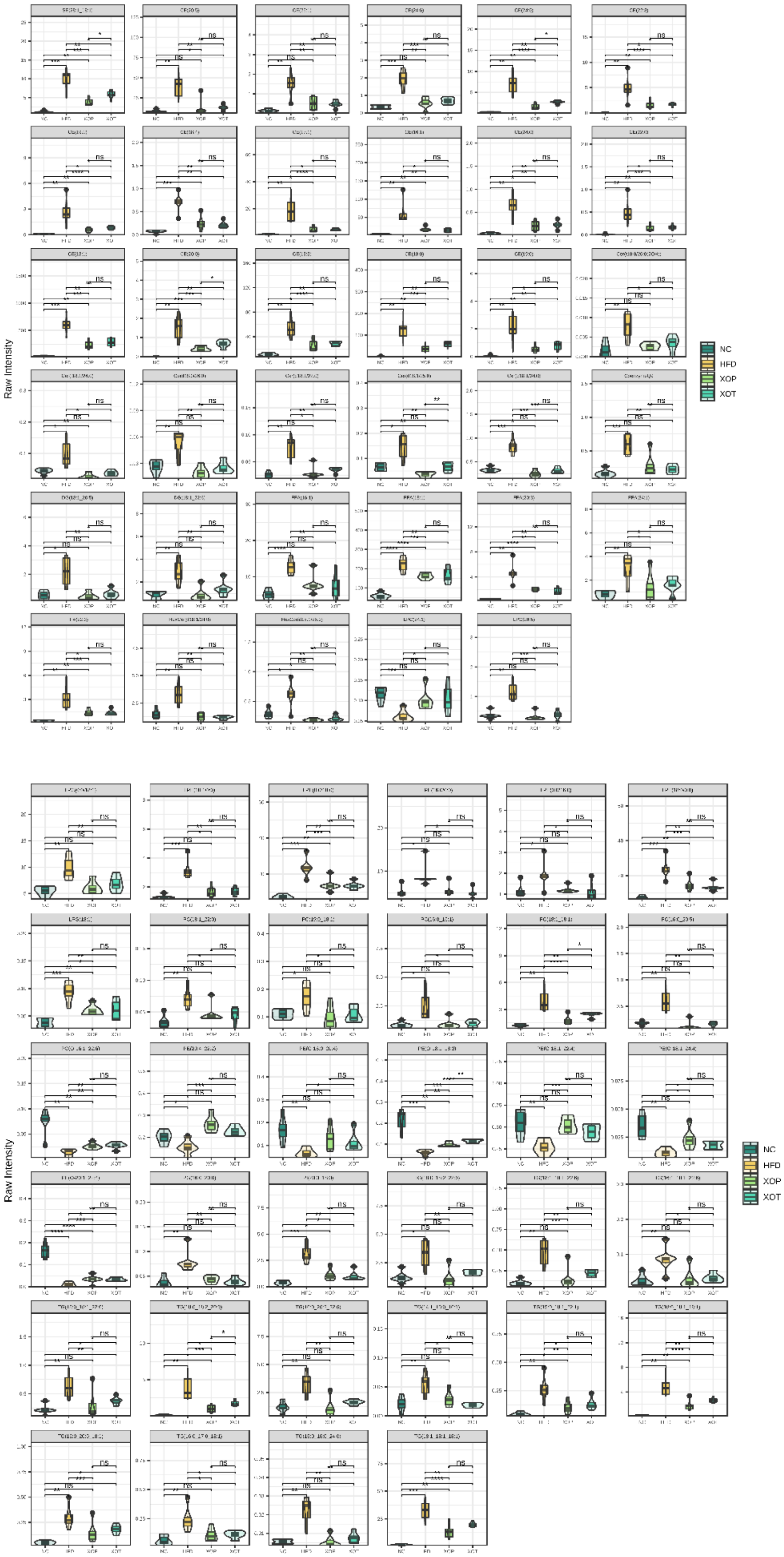

Figure S2. Violin plots of 69 differential serum lipid metabolites in four groups

Table S1. Changes in differential lipid metabolites in the sera of the four groups

Compounds	Primary classification of substances	HFD VS NC	XOP VS HFD	XOT VS HFD
SE(28:1_18:1)	Sterol lipids	up	down	down
CE(24:4)	Sterol lipids	up	down	\
CE(20:5)	Sterol lipids	up	down	down
CE(26:1)	Sterol lipids	up	down	down
CE(24:6)	Sterol lipids	up	down	down
CE(18:2)	Sterol lipids	up	down	\
CE(24:5)	Sterol lipids	up	down	down
CE(22:3)	Sterol lipids	up	down	down
CE(20:2)	Sterol lipids	up	down	\
CE(19:1)	Sterol lipids	up	down	down
CE(18:4)	Sterol lipids	up	down	down
CE(17:1)	Sterol lipids	up	down	down
CE(16:1)	Sterol lipids	up	down	down
CE(24:0)	Sterol lipids	up	down	down
CE(22:0)	Sterol lipids	up	down	down
CE(18:1)	Sterol lipids	up	down	down
CE(20:0)	Sterol lipids	up	down	down
CE(18:3)	Sterol lipids	up	down	down
CE(20:1)	Sterol lipids	up	down	\
CE(18:0)	Sterol lipids	up	down	down
CE(19:0)	Sterol lipids	up	down	down
Cer(t18:0/26:0(2OH))	Sphingolipids	up	down	down
Cer(t18:1/24:0)	Sphingolipids	up	down	down
Cer(t18:0/26:0)	Sphingolipids	up	down	down
Cer(d18:1/22:0)	Sphingolipids	up	down	down
Cer(d18:1/25:0)	Sphingolipids	up	down	down
Cer(d18:1/24:0)	Sphingolipids	up	down	down
CoenzymeQ9	Isoprenol lipids	up	down	down
DG(18:1_20:5)	Glycerolipid	up	down	down
DG(18:1_22:6)	Glycerolipid	up	down	down
DG(18:1_18:1)	Glycerolipid	up	down	\
DG(16:0_18:1)	Glycerolipid	up	down	\
FFA(16:1)	Fatty acyl	up	down	down
FFA(18:1)	Fatty acyl	up	down	down
FFA(20:3)	Fatty acyl	up	down	down
FFA(34:1)	Fatty acyl	up	down	down
FFA(22:3)	Fatty acyl	up	down	down

Continued table.

Compounds	Primary classification of substances	HFD VS NC	XOP VS HFD	XOT VS HFD
HexCer(d18:1/24:0)	Sphingolipids	up	down	down
HexCer(d18:1/25:0)	Sphingolipids	up	down	down
LPC(24:1)	Glycerophospholipids	down	up	up
LPC(20:5)	Glycerophospholipids	up	down	down
LPC(18:1/0:0)	Glycerophospholipids	up	down	\
LPC(0:0/18:1)	Glycerophospholipids	up	down	down
LPE(22:6)	Glycerophospholipids	up	down	\
LPE(18:1/0:0)	Glycerophospholipids	up	down	down
LPE(20:4)	Glycerophospholipids	up	down	\
LPE(0:0/18:0)	Glycerophospholipids	up	down	down
LPE(16:0/0:0)	Glycerophospholipids	up	down	down
LPE(0:0/16:0)	Glycerophospholipids	up	down	down
LPE(18:0/0:0)	Glycerophospholipids	up	down	down
LPG(18:1)	Glycerophospholipids	up	down	down
PC(18:1_22:0)	Glycerophospholipids	up	down	down
PC(19:0_18:1)	Glycerophospholipids	up	down	down
PC(18:0_18:1)	Glycerophospholipids	up	down	\
PC(16:0_16:1)	Glycerophospholipids	up	down	down
PC(16:1_18:1)	Glycerophospholipids	up	down	\
PC(18:1_18:1)	Glycerophospholipids	up	down	down
PC(18:0_18:2)	Glycerophospholipids	up	down	\
PC(16:0_20:5)	Glycerophospholipids	up	down	down
PC(18:0_20:3)	Glycerophospholipids	up	down	\
PC(O-16:1_22:6)	Glycerophospholipids	down	up	up
PE(20:4_22:2)	Glycerophospholipids	down	up	up
PE(22:6_16:0)	Glycerophospholipids	up	down	\
PE(16:0_20:4)	Glycerophospholipids	up	down	\
PE(18:1_18:1)	Glycerophospholipids	up	down	\
PE(18:0_18:2)	Glycerophospholipids	up	down	\
PE(O-16:0_20:4)	Glycerophospholipids	down	up	up
PE(O-18:0_20:4)	Glycerophospholipids	down	up	\
PE(O-16:1_20:4)	Glycerophospholipids	down	up	\
PE(O-18:1_18:2)	Glycerophospholipids	down	up	up
PE(O-18:1_22:4)	Glycerophospholipids	down	up	up
PE(O-18:1_24:4)	Glycerophospholipids	down	up	up
PE(O-20:1_22:4)	Glycerophospholipids	down	up	up
PE(P-15:0_20:3)	Glycerophospholipids	up	down	\
PG(16:0_20:0)	Glycerophospholipids	up	down	down
PG(24:0_20:4)	Glycerophospholipids	down	up	\

Continued table.

Compounds	Primary classification of substances	HFD VS NC	XOP VS HFD	XOT VS HFD
PG(22:0_18:1)	Glycerophospholipids	up	down	\
PI(20:3_18:0)	Glycerophospholipids	up	down	down

Continued table.

Compounds	Primary classification of substances	HFD VS NC	XOP VS HFD	XOT VS HFD
TG(18:0_18:2_22:6)	Glycerolipid	up	down	down
TG(18:1_18:1_22:6)	Glycerolipid	up	down	down
TG(16:1_18:1_22:6)	Glycerolipid	up	down	down
TG(18:0_18:1_22:6)	Glycerolipid	up	down	down
TG(18:1_20:1_22:6)	Glycerolipid	up	down	\
TG(18:0_18:0_22:6)	Glycerolipid	up	down	\
TG(18:0_18:2_20:2)	Glycerolipid	up	down	\
TG(18:0_18:2_20:3)	Glycerolipid	up	down	down
TG(18:0_16:1_24:6)	Glycerolipid	up	down	\
TG(16:0_20:2_22:6)	Glycerolipid	up	down	down
TG(14:1_16:0_16:1)	Glycerolipid	up	down	down
TG(18:0_18:2_20:0)	Glycerolipid	up	down	\
TG(15:0_18:1_22:1)	Glycerolipid	up	down	down
TG(18:0_18:1_18:1)	Glycerolipid	up	down	down
TG(14:0_18:0_20:2)	Glycerolipid	up	down	\
TG(16:0_20:0_18:1)	Glycerolipid	up	down	down
TG(24:0_18:1_18:1)	Glycerolipid	up	down	\
TG(16:0_17:0_18:1)	Glycerolipid	up	down	down
TG(15:0_16:0_24:0)	Glycerolipid	up	down	down
TG(24:0_18:1_20:1)	Glycerolipid	up	down	\
TG(17:1_18:1_20:2)	Glycerolipid	up	down	\
TG(18:1_18:1_18:2)	Glycerolipid	up	down	\
TG(18:1_18:1_18:1)	Glycerolipid	up	down	down
TG(18:1_18:1_19:1)	Glycerolipid	up	down	\
TG(16:0_18:1_18:1)	Glycerolipid	up	down	\
